# Source of Colors

**Published:** 1890-03

**Authors:** 


### SOURCE OF COLORS.

The cochineal insects furnish a great many colors. Among them are carmine, crimson, scarlet-carmine, and purple-lakes. A sea shell belonging to the purpura, and found in Japanese waters, gives a rich violet dye. The cuttlefish gives the sepia. It is the inky fluid which the fish discharges in order to render the water opaque when attacked. Indian-yellow comes from the feces of the camel. Ivory chips produce ivory and bone black. Prussian blue is made by fusing horses' hoofs and other refuse animal matter with impure potasium carbonate. Various lakes are derived from roots, barks, and gums. Lamp-black is soot from certain resinous substances. Turkey red is is made from the madder plant, which grows in Hindoostan. The yellow sap of a tree of Siam produces gamboge; the natives catch the sap in cocoanut shells. Raw sienna is the natural earth from the neighborhood of Sienna, Italy. Raw umber is also an earth found near Umbria, and burnt. India ink is made from burnt camphor and gum. The Chinese and Japanese are the only manufacturers of this ink. The process is a tedious one, and requires great skill. The finer grades of India ink are delicately scented with atter of roses, and one stick about three inches long may cost four or five dollars. Age improves the ink. Mastic is made from the gum of the mastic tree, which grows in the Grecian Archipelago. Bistre is the soot of wood ashes. Very little real ultramarine is found in the market. It is obtained from the precious lapis lazuli, and commands a fabulous price. Chinese white is zinc, scarlet is iodide of mercury, and native vermilion is from the quick-silver ore called cinnabar.—*Medical and Surgical Reporter.*

